# What is helpful and not? Patients’ opinions and beliefs of how to support reduced sedentary behavior during cancer treatment—a thematic analysis

**DOI:** 10.1007/s00520-026-10339-0

**Published:** 2026-01-28

**Authors:** Anna Henriksson, Anne Söderlund, Magnus L. Elfström, Petra von Heideken Wågert

**Affiliations:** 1 School of Health, Care and Social Welfare, Division of Physiotherapy, Mälardalen University, Västerås/Eskilstuna, Sweden; 2 School of Health, Care and Social Welfare, Division of Psychology, Mälardalen University, Västerås/Eskilstuna, Sweden

**Keywords:** Breast cancer, Colorectal cancer, Experience, Focus group, Prostate cancer, Thematic analysis, Sedentary behavior

## Abstract

**Purpose:**

Understanding patients’ opinions and beliefs regarding how to support an active life and decrease sedentary behavior (SB) during treatment is necessary to inform effective interventions for reduced SB. Therefore, the purpose was to explore the opinions and beliefs of patients receiving neo- or adjuvant cancer treatment on how to support the decrease in SB and how to replace sedentary time.

**Methods:**

A purposive sample of patients receiving neo- or adjuvant cancer treatment for breast (*n* = 10), prostate (*n* = 5), or colorectal cancer (*n* = 7) partook in five focus group interviews. Transcripts from the interviews were analyzed with a thematic analysis.

**Results:**

Three themes were identified in the analysis: participants describe that the decrease of SB and replacing time in sedentary behavior can be supported by *Healthcare and employer engagement*, *Socialization*, and lastly by *Self-help*. For instance, early information and encouragement from healthcare personnel, adaptations at work, support from relatives and peers, as well as finding routines and setting goals were considered important for reducing SB. Focusing on daily activities and joyful activities were examples of ways to replace sedentary time.

**Conclusions:**

Interventions for reducing SB may benefit from addressing several aspects from the patient’s life and focusing on replacing SB with daily activities and not only on health-enhancing physical activity. Healthcare staff should provide early information and continuous encouragement for patients about reducing sedentary behavior during oncological treatment. Collaboration with patients to identify and implement practical strategies such as support from family and friends, workplace adaptations, and goal setting may be helpful.

## Introduction

Spending long periods engaged in sedentary behavior (SB), i.e., any waking behavior with a low energy expenditure (≤ 1.5 metabolic equivalents), while in a sitting, reclining, or lying posture [1] may have deteriorating effects for patients with cancer during and after oncological treatment [2]. For instance, an observational study with breast cancer survivors found an association between long bouts of sedentary time (i.e., bouts > 20 min) and lower quality of life in individuals who did not also engage in high levels of moderate to vigorous physical activity [3]. Similarly, a longitudinal study with colorectal cancer survivors found that decreases in prolonged SB (i.e., bouts of > 30 min) and increased moderate to vigorous physical activity were independently associated with better quality of life [4].

An individual can have high levels of SB and still adhere to the physical activity recommendations [5, 6]; however, the physical activity does not necessarily compensate for the SB negative impact on health (e.g., cardiovascular disease mortality or cancer mortality) [7]. Previous interventions focusing on exercise or other health behaviors (including physical activity) have not been effective in also reducing sedentary behavior [8, 9]. This suggests that SB and physical activity may be two distinct behaviors, and other strategies may be needed to target SB [9].

Patients diagnosed with cancer often decrease their physical activity level and increase time in SB, especially during oncological treatment, suggesting that both maintaining low levels of SB and high levels of physical activity are challenging. A study including 199 women with breast cancer found that women with higher levels of fatigue and depressive symptoms were less likely to consistently adhere to physical activity guidelines [10]. Indeed, a previous interview study conducted by our research group found that patients receiving oncological treatment against breast, prostate, or colorectal cancer experience SB as an adjustment to side effects and a strategy to manage cancer-related fatigue [11]. For individuals experiencing high levels of fatigue or other side effects, reducing sedentary time and exchanging it for any intensity physical activity (including low intensity) may be a more feasible approach for individuals during the treatment period. Still, to date, the research focus has been targeting moderate to vigorous physical activity or physical exercise during and after cancer treatment [12], while interventions exclusively focusing on reducing SB are presently fewer for patients with cancer [13]. To date, the intervention studies conducted have mainly been feasibility studies and included multiple intervention components such as combined counseling and fitness trackers. Moreover, most studies have focused on increasing moderate-to-vigorous physical activity rather than specifically targeting reductions in SB, making it unclear how effective these interventions are for reducing SB [13]. Increasing understanding of how to develop effective support to help patients decrease and maintain low levels of SB is therefore warranted.

When designing effective interventions, the patient’s perspective on how to best support is salient. Unfortunately, previous research is lacking qualitative evidence conducted a priori the intervention, which may hamper the success of the intervention. Starting from the “user perspective,” i.e., the patients that need and are to utilize the support, may increase the probability for a successful intervention [14].

To construct effective intervention targeting SB, understanding patients’ opinions and beliefs regarding what kind of support would best help them in decreasing SB and how to replace sedentary time is important. Therefore, the aim of this study was to explore the opinions and beliefs of patients receiving neo- or adjuvant cancer treatment on how to support decrease in sedentary behavior and how to replace sedentary time.

## Method

### Design

This is an explorative qualitative study. We used the Consolidated Criteria for Reporting Qualitative Research checklist for reporting this study [15]. This study was conducted within a postpositivist framework, which suggests that participants’ experiences represent multiple perspectives and not a single reality [16].

### Participants

Persons about to start or undergoing neo- or adjuvant cancer treatment against breast, prostate, or colorectal cancer were eligible for inclusion. A purposeful sample (i.e., individuals with different types of oncological treatments and in different stages of treatment) of patients with breast (*n* = 10), prostate (*n* = 5), and colorectal cancer (*n* = 7) undergoing neo- or adjuvant cancer treatment was included (Table 1). Exclusion criteria were advanced cancer, dementia, severe psychiatric disease, or a severe communicative disability. Potential participants were identified and provided with written and oral information regarding the study adjacent to visiting a university hospital located in Sweden (either provided by a researcher during the visit or posted home to the patients after the visit). The individuals were contacted via telephone a few days later and asked for participation.

**Table 1:** Participants characteristics (*n* = 22)

Diagnosis	
Breast cancer, *n* (%) Prostate cancer, *n* (%) Colorectal cancer, *n* (%)	10 (45) 5 (23) 7 (32)
Age, years mean (min–max)	69.9 (31–79)
Sex	
Women, *n* Men, *n*	12 10
Cancer treatment type^1^	
Neo- or adjuvant chemotherapy, *n* Neo- or adjuvant antibody treatment, *n* Adjuvant radiotherapy, *n* Adjuvant endocrine treatment, *n* Surgery^2^, *n*	13 3 10 9 17
Education (missing *n* = 2)	
High school, *n* University or college, *n*	7 11
Current work status	
Working 100–50%, *n* Sick leave 100–50%, *n* Retired, *n*	12 8 9
Living situation	
Living with spouse/partner, *n* Has children, *n*	15 18
Financial situation (scale 0–10)^2^	
Median (max–min)	6 (9–3)
Self-reported time spent sedentary^3^	
Mean hours/day (max–min)	6.6 (11.0–2.5)
Confidence in reducing time spent sedentary (scale 0–10)^4^	
Median (max–min)	5.5 (10–0)

^1^Participants could have one or a combination of cancer treatments

^2^A scale where 0 = “Worst possible” and 10 = “Best possible” financial situation

^3^Participants answered the question: “During your cancer treatment period, how much time do you spend sitting and lying down during the day?” and responded by reporting the total hours and minutes

^4^Participants answered the question: “How confident are you in reducing sedentary behavior during cancer treatment?” and responded on a Likert scale where 0 is “Not at all confident” and 10 “Absolutely confident” in reducing time spent sedentary during cancer treatment period

### Data collection

Prior to the interviews, the participants signed a consent form and completed a questionnaire containing study-specific questions regarding sex, age, type of cancer treatment, education, current work status, living situation, financial situation, and SB, that we previously used in a qualitative study [11]. SB was assessed with the following questions: “During your treatment period, how much time do you spend sitting and lying down during the day?” and “How confident are you in reducing sedentary behavior during cancer treatment?” (Table 1). The consent form and questionnaire were posted back to the researcher with a pre-paid envelope.

Participants were interviewed in five focus groups (total *n* = 22), which lasted between 52 and 78 min. Group size ranged from 3 to 5 individuals in each group. We continued data collection until no new information emerged from the focus groups. Focus groups were chosen as the data collection method because the interaction between participants can enrich the data collection [17]. Due to the COVID-19 pandemic, the focus groups were conducted via a web-based video platform (Zoom, Inc.); however, the focus groups were recorded on a digital audio recorder, and the only data collected were the audio and researcher’s (first authors’) notes. The participants were encouraged to find a secluded space free of interference for the interview, and all participants joined the focus group from their homes. During the focus groups, a semi-structured interview guide was used to ensure that all discussion points were covered in all groups yet allowed for probing questions by the interviewer if necessary (Table 2). The role of the interviewer (first author—a registered nurse and researcher) was to moderate, stimulate, and focus the discussions. The researchers were not involved in the participants’ cancer care.

**Table 2:** Interview guide

Question areas	
Support for reduced sedentary behavior *Can you tell me how YOU think one can best support to reduce sedentary behavior when receiving treatment?* Replace time in a sedentary position Can you tell me what YOU think is the best way to replace sedentary time? Support to maintain reduced sedentary time *Can you tell us how YOU think you can best maintain reduced sedentary behavior?* In addition to these questions, clarifying questions and follow-up questions were asked, such as “can you tell me more about it?” and “what do you think/feel about it?”	

### Analysis

A six-step thematic analysis was conducted as described by Braun and Clarke to identify patterns or themes in the data set [18]. Interviews were transcribed verbatim and then checked against the recording for accuracy.

The step-wise analysis was made with an inductive approach, and the analysis was aimed at identifying semantic themes. The inductive approach was chosen due to the limited existing knowledge about how patients’ opinions and beliefs influence their support needs to reduce sedentary behavior. This approach allowed themes to arise directly from participants’ accounts rather than being shaped by a predetermined theoretical model.

In the first step of the analysis, the transcribed interviews were read several times to familiarize with the data, and initial ideas about what the data contained were noted by the first author. In the second step of the analysis, initial codes were generated by identifying text extracts that corresponded to the purpose of the study and labeling them with short semantic descriptions of the meaning. An extract could hold several codes (Table 3). Coding was conducted systematically and iteratively. The first author constructed the initial codes, which were discussed with all the co-authors regularly to support reflexivity and enhance interpretive consistency. The third step entailed sorting the codes into potential themes and sub-themes and then creating a thematic map over the identified themes and sub-themes; again, the first author constructed suggestions that were discussed with the co-authors. Then, in the fourth step of the analysis, the themes and sub-themes were checked against the text extracts and the entire data set. The fifth and sixth steps entailed defining and naming the themes, selecting text extracts to exemplify the themes, and writing the results. The fourth and fifth steps were again conducted through several sessions where all the authors discussed and revised the themes. All analytic decisions were documented to ensure an audit trail.

**Table 3:** Examples of data analysis

Data extracts	Initial codes	Sub-themes	Themes
Group 1, Participant 1: “It’s good that you get support. That they inform… [health care workers]. The sooner the better. I think that’s the melody. Then it may be that you need to nag about it, because what you hear the first time- you forget. Or at least I did…”	Early support Repeated information	Health care personnel providing early repeated and accurate information	Healthcare and employer engagement
Group 4, Participant 2 “My grandchild took me out and we searched, what is it called… Pokemon-figures from the movies… He had an app with a map and then you had to walk around and search… […] It was very fun to be together and do something… You almost forgot that you were getting treatment.”	Someone to take me out	Family, friends and colleagues pushing	Socialization
Group 5, Participant 4 “I always take a walk every morning […] even during treatment, although much shorter [distance]. Its nice to have it done in the morning, then you don’t have to worry about it later.”	Daily routines	Finding routines	Self-help

## Results

**Fig. 1:**
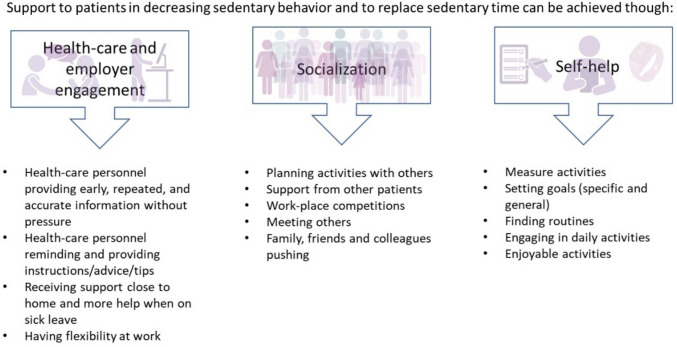
Overview of themes and sub-themes

The thematic analysis resulted in 3 themes and 14 sub-themes (see Fig. 1 for an overview of themes and sub-themes). The three themes describe that the decrease of SB and replacing sedentary time can be supported in the short and long term by *Healthcare and employer engagement*, *Socialization*, and lastly by *Self-help*.

### Healthcare and employer engagement

The participants believed that receiving support from healthcare personnel was important from different aspects, such as providing information about the importance of reducing SB and increasing physical activity. It was considered important to receive this information early in the treatment trajectory, and the information needed to be repeated. For instance, participants described the value of receiving information before or soon after the treatment started, which was that they then had not yet experienced the side effects of the treatment. They therefore believed it would be more feasible to be active and easier to motivate patients. Yet, participants also highlighted that it was not certain that they were receptible to the information at that time, which is why information also needed to be repeated and patients reminded throughout the treatment trajectory and not just once at the start of treatment.

Further, having personnel ask about daily activities when the patient comes to the clinic for a planned chemotherapy treatment was considered helpful, because it served as a reminder, and emphasized that “this is important,” yet without creating negative pressure. “There is no pressure that you have to perform anything, just that it is good if you go out and take a walk” (Group1, Participant 2). Therefore, the participants thought that visits to the hospital for receiving treatment or follow-ups were considered good opportunities for being reminded to be active. “So when I was in and had my chemotherapy they [healthcare personnel] talked quite a lot about general well-being. How you felt and what you did during the days, and stuff like that. And reminded that: ‘yes, but it is good that you go out and take a walk’” (Group1, Participant 2).

The accuracy as well as tailoring the information to the patient’s situation and physical ability was also emphasized as important. Failure to do so could lead to increased experience of stress; for instance, a participant described receiving advice regarding physical activity during the treatment that was difficult to achieve: “Then they [the nurses] told me to exercise for an hour every day. It was very difficult for me to achieve that, because I had the children to look after and stuff like that too… And after an hour I was very tired. Then my whole day was ruined because I was too tired for anything else” (Group2, Participant 4).

Other support that participants wished for was healthcare personnel providing them with instructions and practical advice about what activities they could perform.

Participants expressed that being able to work may have been important for staying active during the treatment. Additionally, participants expressed that having support from their employer and workplace was considered important. For instance, having a manager who was supportive by providing an adapted work environment that allowed for individuals to work during treatment, but also allowing for going to exercise classes during work hours, was considered helpful. Examples of adaptations could be allowing for different work hours or flexibility regarding working at the office or at home. However, it was also recognized by the participants that this was not possible for all types of work and that individuals who need to be on sick leave may need to receive extra support.

### Socialization

Planning activities with others was considered a helpful strategy to reduce SB. For instance, having regularly scheduled meetings for walking or other activities was described as decreasing the barriers to being active because the social interaction provided both motivation and social pressure to honor previously agreed upon commitments. “…*You can have fixed meetings maybe every 14:th day or once a week or something and then we do this. […] you can go out for a walk or something like that. So that you find something to do*” (Group 1, Participant 2).

The social support could be provided from several sources: family members, friends, or colleagues as well as other patients with cancer. “I think many need support from others. Because sometimes they feel very ill…” (Group 2, Participant 2)*.* Participants thought it was an advantage to receive support from individuals that you interact with daily (i.e., family, friends, colleagues), but also support provided by peers was considered helpful because the support would be provided by someone who could relate to their situation. “They need support from someone who has a similar situation and actually understands and can be supportive” (Group 2, Participant 2).

The support could consist of different ways to interact, such as having friendly activity competitions at work or partaking in exercise groups or walking groups for cancer survivors. Participants suggested that “just meeting others” could be helpful to stay active and “get one out of the house.” Also, having someone asking you to come along, even when the participants were experiencing side effects such as fatigue, could be helpful. One participant described that the side effects could make one passive and not be able to come up with something to do, and having a partner asking or suggesting activities may be key for staying active.

### Self-help

Participants suggested different helpful self-help strategies to stay active and reduce SB during treatment. Measuring and keeping track of activities was believed to be helpful because it increased awareness of how much an individual was active; however, it was also important to combine this with a goal. Participants gave examples of mobile applications that keep track of time spent sedentary, count steps, time spent in physical activities, and walking distance: “… there are apps, this one with steps, that you decide that now I’m going to walk so many steps” (Group 5, Participant 4). Some of these applications had a goal-setting function that clearly showed how the individual progressed; however, even though this feature was considered helpful, it was not necessary for all participants for keeping track of their goal achievements: “So that it is important that you have something like ‘this is good and now I have fulfilled it’” (Group 5, Participant 4).

Additionally, using technology that, besides keeping track of activities, also provided reminders to break up prolonged sitting time was a desirable feature.

To stay active and not become more sedentary during treatment was considered important to find routines that were adapted to the individual’s situation. For instance, even if the goals of activities (e.g., such as walking a daily number of steps) may change due to side effects, keeping the routine (e.g., to take daily walks) was considered important. Other examples of routines participants described were “always walking around when speaking on the phone” or “taking a break from sitting down every hour when at work.” Furthermore, engaging in daily activities and activities that were joyful was helpful because the participants believed it increased the chance of staying active. “I also really believe in this, that it should be something you can do often and think is fun… working in the garden in the summer… that's also good [for you]… and then you get to be outdoors” (Group 3, Participant 1).

## Discussion

The themes identified in this study suggest that the support wanted by patients undergoing cancer treatment can and should be provided at three levels: at the community level, including support provided from healthcare professionals and the workplace; at an interpersonal level, receiving support from family, colleagues, and friends; and finally, the support aiming at helping the individual increase his or her self-management ability. Consistent with previous research conducted with a focus on physical activity and cancer [19], the participants emphasized the importance of the role of the healthcare personnel in providing encouragement and advice. Receiving early information from healthcare personnel, even at the time of diagnosis, may be important. A study investigating the preferences of newly diagnosed women planned for cardiotoxic chemotherapy against breast cancer found that 61.9% of the women wanted information regarding exercise programs at the time of diagnosis [20]. However, the results from this present study also indicate that only providing standardized information regarding physical activity (e.g., recommendations of health-enhancing physical activity) is not helpful for patients struggling with keeping active, i.e., the support needs to be adapted to the patients’ current fatigue level and any co-morbidities [13]. Our results support the importance of patient-centered support because failure of healthcare personnel to provide patient-centered advice (e.g., providing advice not adapted to the patients’ experienced fatigue or unrealistic for the patient) may negatively affect the patient. The participants also described that they wanted support close to home. These results suggest that interventions may need to be digitally adapted or conducted within primary care for individuals with long travel distances to the treatment center.

The results also suggest that it is important to tailor support based on the individual’s work status. A study investigating determinants of physical activity and SB in 175 participants after treatment for breast, colorectal, lung, and prostate cancer similarly identified that being on sick leave or retired was associated with higher levels of physical activity than in patients working [21]. In addition, our results suggest that the type of work and the adaptability of the work are also important to consider when supporting reduced SB. Furthermore, the work place seems to be important also on an intrapersonal level for receiving support from colleagues. Thus, the workplace may be an additional arena for interventions aiming to reduce SB for cancer survivors.

Similar to research conducted for patients living with obesity [22], our participants described the importance of support from others, including family, work colleagues, and other patients (i.e., peer support) for reducing sedentary behavior. Also, for patients treated against cancer, perceived strong social support has been associated with higher physical and psychological health-related quality of life [23].

The participants described strategies that are known effective behavior change techniques (i.e., goal setting, self-monitoring, reminders) for increasing physical activity for cancer survivors [24], suggesting that these may also be helpful in reducing SB. Using technology containing, e.g., self-monitoring behavior change techniques such as activity monitors or mobile applications, was also considered helpful. A systemic review [13] for reducing sedentary behavior in cancer patients and survivors showed five of nine included studies used either activity monitors, text messages, or applications. Most of the studies were feasibility studies; however, the authors identified that there is an overall trend where intervention groups decreased SB in comparison to controls, suggesting that these are promising strategies [13].

In addition, the results highlight the importance of daily activities and choosing enjoyable activities for being motivated to reduce SB. Therefore, interventions may benefit from letting the participants choose the activity they could replace the sedentary time with. Another strategy described by participants was to find and/or maintain routines throughout the oncological treatment. To maintain an activity routine (e.g., going for a walk) during the treatment period, it is important that there is also flexibility in, for example, how intense the activity is (brisk or slow walk) so that the activity can be adapted with regard to side effects. 

From a clinical practice perspective, our findings highlight that healthcare professionals can play a key role by providing early and clear information as well as continuous follow-up throughout the treatment period. Additionally, support can be offered by helping patients identify practical and achievable ways to incorporate more movement into their daily routines. Professionals can also encourage patients to involve family or friends for reminders and motivation, make adaptations to workplace routines, and set personalized goals to reduce sedentary behavior.

### Strengths and limitations

The focus groups were conducted virtually due to the restrictions imposed by the COVID-19 pandemic. Although there is limited research investigating the differences between in-person and virtual focus groups, conducting them virtually may have some potential benefits, such as eliminating the need for participants to travel and being more feasible for individuals with health issues [25]. Several steps were taken to ensure trustworthiness [26] of this study. For instance, the dependability was enhanced by having an experienced interviewer and by utilizing an interview guide. An additional strength was the multi-professional composition of the author group (a nurse, two physiotherapists, and a psychologist) and their previous extensive experience with qualitative research, which enhanced the credibility of this study. Also, a systematic use of Braun and Clarke’s described thematic analysis [18] and comprehensive discussions between the authors throughout the entire analysis process were important to increase confirmability.

A limitation of this research was that a higher proportion of our participants had a university degree, which has also been the case in previous research on physical activity for patients with cancer [27] and an interview study regarding the experience of SB during oncological treatment [11]. It also stands to reason that individuals participating in this study may be inclined to have a favorable attitude toward physical activity and reducing sedentary time, i.e., high health literacy [28]. It may be that individuals feeling more negatively toward reducing SB experience different barriers and may need other support. Therefore, the transferability of the results may be limited for individuals with lower socioeconomic status and limited health literacy.

## Conclusion

Interventions for reducing SB may benefit from incorporating support from several sources of the individual’s life that includes support provided at a community level, at an interpersonal level, and support for self-management. Similar behavior change techniques used for increasing physical activity may be important for reducing SB. Also, focusing on replacing sedentary time with daily activities and not only focusing on health-enhancing physical activity may be a helpful strategy for staying active through cancer treatment.

## Data Availability

Pseudonymised data are available from the corresponding author on reasonable request.

## References

[1] Tremblay MS, Aubert S, Barnes JD et al (2017) Sedentary behavior research network (SBRN) – terminology consensus project process and outcome. Int J Behav Nutr Phys Act 14:75. 10.1186/s12966-017-0525-8 28599680 10.1186/s12966-017-0525-8 PMC5466781

[2] Swain CTV, Nguyen NH, Eagles T et al (2020) Postdiagnosis sedentary behavior and health outcomes in cancer survivors: a systematic review and meta‐analysis. Cancer 126:861–869. 10.1002/cncr.32578 31714596 10.1002/cncr.32578

[3] Hartman SJ, Nelson SH, Myers E et al (2017) Randomized controlled trial of increasing physical activity on objectively measured and self-reported cognitive functioning among breast cancer survivors: the memory & motion study: physical activity and cognition RCT. Cancer. 10.1002/cncr.30987 28926676 10.1002/cncr.30987 PMC5735009

[4] Kenkhuis MF, Van Roekel EH, Breedveld-Peters JJL et al (2021) Longitudinal associations of sedentary behavior and physical activity with quality of life in colorectal cancer survivors. Med Sci Sports Exerc 53:2298–2308. 10.1249/MSS.0000000000002703 34033619 10.1249/MSS.0000000000002703 PMC8542069

[5] Bull FC, Al-Ansari SS, Biddle S (2020) World Health Organization 2020 guidelines on physical activity and sedentary behaviour. Br J Sports Med. 10.1136/bjsports-2020-102955 33239350 10.1136/bjsports-2020-102955 PMC7719906

[6] Campbell KL, Winters-Stone KM, Wiskemann J et al (2019) Exercise guidelines for cancer survivors: consensus statement from international multidisciplinary roundtable. Med Sci Sports Exerc 51:2375–2390. 10.1249/MSS.0000000000002116 31626055 10.1249/MSS.0000000000002116 PMC8576825

[7] Ekelund U, Brown WJ, Steene-Johannessen J (2019) Do the associations of sedentary behaviour with cardiovascular disease mortality and cancer mortality differ by physical activity level? A systematic review and harmonised meta-analysis of data from 850 060 participants. Br J Sports Med. 10.1136/bjsports-2017-098963 29991570 10.1136/bjsports-2017-098963

[8] Lynch BM, Courneya KS, Sethi P et al (2014) A randomized controlled trial of a multiple health behavior change intervention delivered to colorectal cancer survivors: effects on sedentary behavior. Cancer 120:2665–2672. 10.1002/cncr.28773 24816611 10.1002/cncr.28773

[9] Pinto B, Dunsiger S, Stein K (2017) Does a peer-led exercise intervention affect sedentary behavior among breast cancer survivors? Psychooncology 26:1907–1913. 10.1002/pon.4255 27531024 10.1002/pon.4255 PMC5581246

[10] Brunet J, Amireault S, Chaiton M, Sabiston CM (2014) Identification and prediction of physical activity trajectories in women treated for breast cancer. Ann Epidemiol 24:837–842. 10.1016/j.annepidem.2014.07.004 25174285 10.1016/j.annepidem.2014.07.004

[11] Henriksson A, Elfström ML, Söderlund A, von Heideken Wågert P (2024) Exploring sedentary behavior during neo-or adjuvant treatment in patients with cancer: a phenomenological study. Eur J Oncol Nurs 70:102556 38636117 10.1016/j.ejon.2024.102556

[12] Patel AV, Friedenreich CM, Moore SC et al (2019) American College of Sports Medicine roundtable report on physical activity, sedentary behavior, and cancer prevention and control. Med Sci Sports Exerc 51:2391–2402. 10.1249/MSS.0000000000002117 31626056 10.1249/MSS.0000000000002117 PMC6814265

[13] Belcher BR, Kang D-W, Yunker AG, Dieli-Conwright CM (2022) Interventions to reduce sedentary behavior in cancer patients and survivors: a systematic review. Curr Oncol Rep 24:1593–1605. 10.1007/s11912-022-01313-0 35829982 10.1007/s11912-022-01313-0

[14] Bombard Y, Baker GR, Orlando E et al (2018) Engaging patients to improve quality of care: a systematic review. Implement Sci 13:98. 10.1186/s13012-018-0784-z 30045735 10.1186/s13012-018-0784-z PMC6060529

[15] Tong A, Sainsbury P, Craig J (2007) Consolidated criteria for reporting qualitative research (COREQ): a 32-item checklist for interviews and focus groups. Int J Qual Health Care 19:349–357. 10.1093/intqhc/mzm042 17872937 10.1093/intqhc/mzm042

[16] Creswell JW, Poth CN (2017) Qualitative inquiry and research design: choosing among five approaches, 4th edn. Sage publications, Los Angeles

[17] McLafferty I (2004) Focus group interviews as a data collecting strategy. J Adv Nurs 48:187–194. 10.1111/j.1365-2648.2004.03186.x 15369499 10.1111/j.1365-2648.2004.03186.x

[18] Braun V, Clarke V (2006) Using thematic analysis in psychology. Qual Res Psychol 3:77–101. 10.1191/1478088706qp063oa

[19] McDonough MH, Beselt LJ, Kronlund LJ et al (2021) Social support and physical activity for cancer survivors: a qualitative review and meta-study. J Cancer Surviv 15:713–728. 10.1007/s11764-020-00963-y 33128705 10.1007/s11764-020-00963-y

[20] Sturgeon KM, Fisher C, McShea G et al (2018) Patient preference and timing for exercise in breast cancer care. Support Care Cancer 26:507–514. 10.1007/s00520-017-3856-8 28840334 10.1007/s00520-017-3856-8

[21] Aumaitre A, Gagnayre R, Foucaut A-M (2024) Determinants and factors of physical activity and sedentary behaviors among post-treatment breast, colorectal, lung, and prostate cancer survivors living in France: results from the DEFACTO study first phase. Patient Educ Couns 124:108273 38598865 10.1016/j.pec.2024.108273

[22] Curran F, Brennan C, Matthews J, O’ Donoghue G (2024) A qualitative study of perceived barriers and facilitators to interrupting sedentary behavior among adults living with obesity. Obes Sci Pract 10:e721. 10.1002/osp4.721 38263998 10.1002/osp4.721 PMC10804343

[23] Leung J, Pachana NA, McLaughlin D (2014) Social support and health-related quality of life in women with breast cancer: a longitudinal study. Psychooncology 23:1014–1020. 10.1002/pon.3523 24700668 10.1002/pon.3523

[24] Qiu L, Ye M, Tong Y, Jin Y (2023) Promoting physical activity among cancer survivors: an umbrella review of systematic reviews. Support Care Cancer 31:301. 10.1007/s00520-023-07760-0 37097500 10.1007/s00520-023-07760-0 PMC10129958

[25] Rupert DJ, Poehlman JA, Hayes JJ et al (2017) Virtual versus in-person focus groups: comparison of costs, recruitment, and participant logistics. J Med Internet Res 19:e80. 10.2196/jmir.6980 28330832 10.2196/jmir.6980 PMC5382259

[26] Lincoln YS, Guba EG (1985) Naturalistic inquiry. Sage Publications, Beverly Hills, Calif

[27] Henriksson A, Arving C, Johansson B et al (2016) Perceived barriers to and facilitators of being physically active during adjuvant cancer treatment. Patient Educ Couns. 10.1016/j.pec.2016.01.019 26860549 10.1016/j.pec.2016.01.019

[28] Ryman C, Warnicke C, Hugosson S et al (2024) Health literacy in cancer care: a systematic review. Eur J Oncol Nurs. 10.1016/j.ejon.2024.102582 38608377 10.1016/j.ejon.2024.102582

